# Risk of secondary surgery following surgical treatment of fractures: a nationwide register study on 9,719 adult patients

**DOI:** 10.2340/17453674.2025.43446

**Published:** 2025-04-14

**Authors:** Anders Bo ROENNEGAARD, Signe Steenstrup JENSEN, Peter Toft TENGBERG, Per Hviid GUNDTOFT, Bjarke VIBERG

**Affiliations:** 1 Department of Orthopedic Surgery and Traumatology, Kolding Hospital (part of Hospital Lillebaelt), Kolding; 2 Department of Regional Health Research, University of Southern Denmark, Odense; 3 Department of Orthopedic Surgery, Hvidovre Hospital; 4 Department of Orthopedic Surgery, Aarhus University Hospital, Aarhus; 5 Department of Orthopedic Surgery and Traumatology, Odense University Hospital, Odense; 6 Department of Clinical Research, University of Southern Denmark, Odense, Denmark

## Abstract

**Background and purpose:**

Reports on the risk of secondary surgery in fracture-related surgery are scarce in the literature. The aim of this study was to estimate the risk of any secondary musculoskeletal surgery within 2 years of primary, fracture-related surgery.

**Methods:**

We performed a nationwide register study on adult Danish patients surgically treated for fractures in 2016 with 2 years’ follow-up. We used cross-linked data from the Danish Fracture Database, the Danish National Patient Registry and the Danish Civil Registration System. Primary outcome was risk of secondary surgery calculated by the cumulative incidence function and presented with 95% confidence intervals (CI) overall and stratified on age, sex, and anatomical area.

**Results:**

We included 9,719 adult patients of whom 63% were female and median age was 70 years (20–100). The overall risk of secondary musculoskeletal surgery in the same anatomical area as the primary was 20% (CI 19–21), for reoperation (i.e., pertaining to the initial treatment) 19% (CI 18–20), and for major reoperation (due to complication of the initial treatment) 8% (CI 7–8). Across anatomical areas risk ranged from 4% (CI 1–9) to 69% (CI 66–73) for secondary surgery, from 4% (CI 1–9) to 68% (CI 65–72) for reoperations, and from 2% (CI 0–6) to 26% (CI 19–33) for major reoperation.

**Conclusion:**

The risk of experiencing a major postoperative complication that needs surgical treatment is below 10%.

Although fracture-related surgery is often necessary in order for patients to avoid disabilities and chronic pain, it also carries with it the risk of 1 or more secondary surgeries. These can span simple hardware removal carried out after successful healing of the bone to large reoperations due to complications such as infection or hardware failure [1-3]. Recently, more studies considering reoperations after fracture-related surgery have been emerging. In 2021, Pincus et al. [4] found that 20% of more than 45,000 primarily surgically treated ankle fractures, experienced at least 1 subsequent reoperation within 2 years. In a Swedish study concerning proximal humerus fractures, the risk of reoperation was 17% following primary surgery [5]. Most existing studies concern specific types of fractures or primary surgical techniques [6-8]. Moreover, population sizes are often limited. As such, large population studies considering overall secondary surgery in fracture-related surgery in general, including fracture sites other than the ones of major interest such as hip fractures, are warranted.

The aim of this study was to estimate the absolute risk of secondary, musculoskeletal surgery within 2 years of primary, fracture-related surgery.

## Methods

### Study design

In this nationwide register study, we estimated the risk of secondary, musculoskeletal surgery in the same anatomical area 2 years following primary, fracture-related surgery at Danish hospitals in 2016. We estimated risk overall and stratified on anatomical areas for primary surgery. For overall estimates, we furthermore stratified on sex and age group.

Reporting was undertaken according to the RECORD guidelines for observational studies using routinely collected data [9].

### Setting

The healthcare system in Denmark is tax funded. This means that Danish residents enjoy equal access to all levels of public hospital care and general practitioners [10]. Residents are assigned a 10-digit, unique personal identification number. This number is used in all registrations in both private and public health care and in all nationally implemented health care registers [11].

### Data sources

We cross-linked cases from 3 different databases on an individual level by using the personal identification number in order to obtain data on patients.

#### The Danish Fracture Database (DFDB)

The DFDB was established in 2011 with the purpose of gathering prospective data on fracture-related surgeries [12]. It was implemented nationally in 2015. It closed in 2020 containing over 100,000 cases of fracture-related surgery, both primary and planned secondary surgery. Although validity of data in the DFDB has been shown to be high, with positive predictive values ranging from 81% to 100%, the completeness of the DFDB in 2016 was only 55%, meaning that nearly half of fracture-related procedures carried out at public Danish hospitals were not included in the current study [13]. The completeness of reoperations in the DFDB is considered very low. As such, the Danish National Patient Registry (DNPR) is ideal for identifying secondary procedures from the cohort of primary, fracture-related surgical cases from the DFDB.

#### The DNPR

Nationally implemented in 1978, the DNPR covers 99.4% of all hospital discharges in Denmark [14]. Reporting is mandatory for all hospitals in Denmark. Diagnosis codes are registered according to the International Statistical Classification of Diseases version 10 (ICD-10) and procedure codes according to the Nordic Medico-Statistical Committee (NOMESCO) classification system [15,16]. The DNPR provides complete registrations of all diagnosis codes and surgical procedure codes registered at Danish hospitals [14].

#### The Danish Civil Registration System (CPR)

The CPR was established in 1968 and registers all individuals in Denmark for public administration purposes. It contains personal identification numbers on all individuals who are either born to a mother registered in the CPR or have resided in the country for 3 months or more. Reporting is mandatory for all public institutions and the register is recognized as valid and has a completeness of 99.7% [11]. The CPR contains data on exact time of death and emigrations for all residents in Denmark registered with the personal identification number.

### Data collection and study population

Inclusion of patients is shown in the Figure. Only adult patients were included. Adulthood was defined as 20 years of age or older. Besides patients registered with reoperation in the DFDB, patients registered with planned secondary surgery in the DFDB were also excluded, as no information on anatomical site of fracture or surgery was available. Patients registered with multiple fractures or as received by trauma call were also excluded as they represent a separate group of patients when considering risk of secondary surgery. Only first occurrence of primary surgery in the DFDB and first occurrence of any secondary, musculoskeletal surgery in the DNPR were included for analysis in order to maintain independence of observations. Technical data and demographics on primary surgeries were obtained from the DFDB. Data on death and emigration was obtained from the CPR. All data was stored on secure servers hosted by the Danish Health Data Authority in a secure system called “Forskermaskinen.”

**Figure:**
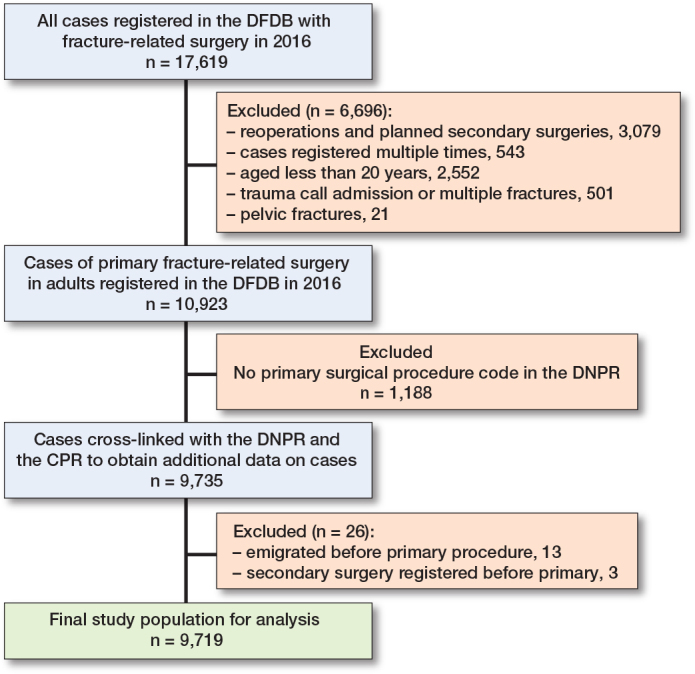
Flowchart of inclusion of patients for study analysis.

### Outcomes

#### Primary outcomes

The primary outcome was risk of secondary musculoskeletal surgery and reoperation. Secondary musculoskeletal surgery was defined as surgical procedure with code N* in the same anatomical area as the primary, fracture-related surgery up to 2 years following the primary surgery.

These surgical procedure codes denoted any kind of musculoskeletal surgery and, as such, did not necessarily pertain to the primary fracture surgery (i.e., NFQ* denoting amputation of the hip or thigh registered after primary registration of fracture-related surgery in the hip or thigh, such as NFJ*). Of the secondary surgeries identified, reoperation was defined as surgeries most likely pertaining to the primary, fracture-related surgery. Reoperation was defined as occurrence of the following surgical procedure codes up to 2 years following primary surgery and could be considered a subgroup of secondary surgery: primary prosthetic replacement (NxB*); secondary prosthetic replacement (NxC*); excision, reconstruction, and fusion of joint (NxG*); fracture surgery, i.e., periprosthetic or implant fracture or re-osteosynthesis (NxJ*); operations on bone, i.e., non-union surgeries (NxK*); amputation and related operations (NxQ*); operations for infection of tendons, joints, and bone (NxS*); removal of implants and external fixation devices (NxU*), and reoperations (NxW*).

Surgical codes denoting surgeries of the skin (Q*), including wound revisions, were not considered in this study.

#### Secondary outcome

Secondary outcome was major reoperation defined as any reoperation within 2 years; however, surgeries registered with NxU* surgical procedure codes were only considered major reoperation under specific circumstances. Removal of arthroplasties was always considered a major reoperation. For osteosynthesis besides K-wires and external fixations, hardware removal was defined as a major reoperation if it occurred less than 12 weeks following primary surgery. Removal of K-wires within 3 weeks following primary surgery was also defined as a major reoperation. For external fixation, hardware removal in combination with a new external or internal fixation (NxJ2*–NxJ9*) within 3 weeks of primary surgery was considered final surgery and not a major reoperation. For external fixation of the lower extremity that had not been replaced within 3 weeks, external fixation was considered primary and final treatment. For these, hardware removal less than 12 weeks following primary surgery was defined as major reoperation. For external fixation of the upper extremity, removal of an external fixator within 6 weeks was considered a major reoperation.

### Outcome measure

Risk was estimated using the cumulative incidence function. Death was a competing risk. Risk was estimated for primary and secondary outcomes overall as well as by sex and age group. Furthermore, it was estimated by anatomical area of primary surgery defined by primary fracture data in the DFDB.

### Missing values

We found missing values for laterality of surgery in secondary surgeries registered in the DNPR. All linkage between primary surgery and secondary surgeries was performed using the personal identification numbers without considering laterality for secondary surgery. A sub-analysis was performed to estimate the issue of not matching laterality on the overall results. No missing values were seen in other variables included in the study from the DNPR, the DFDB, or the CPR.

### Statistics

All statistical analyses were carried out in STATA version 17 (StataCorp LLC, College Station, TX, USA) [17].

We used descriptive statistics for demographic data. Risk of reoperation was estimated as absolute risk using the cumulative incidence function [18]. Death was a competing event and emigration was censoring. Time of inclusion was date for primary surgery in all cases, which meant no left truncation occurred. Follow-up was 2 years from date of primary surgery for all patients.

### Ethics, funding, and disclosures

The study was approved by the Danish Patient Safety Authority (case no. 3-3013-2729/1) and the Danish Data Protection Agency (jr. no. 18-31360). Approval from the Danish National Committee on Health Research Ethics was not necessary as this study did not perform any interventions, nor did it have any patient contact.

The authors report no conflicts of interests. Complete disclosure of interest forms according to ICMJE are available on the article page, doi: 10.2340/17453674.2025.43446

## Results

### Demographic data

Of the 9,719 patients included for analysis, 6,122 were female (63%) and the overall median age was 70 years (20–100). Stratified on anatomical fracture sites, the population ranged from 88 (1%) patients with patellar fractures to 3,931 (40%) patients with hip fractures.

### Risk of secondary surgery, reoperation, and major reoperation

The overall risk of any secondary, musculoskeletal surgery within 2 years of primary, fracture-related surgery was 20% (CI 19–21) (Table). For reoperation it was 19% (CI 18–20) and for major reoperation it was 8% (CI 7–8). Males had a higher risk of experiencing any of the 3 outcomes compared with females. By age, patients aged 20–39 at the time of primary surgery had a higher risk of experiencing secondary surgery and reoperation. Stratified on anatomical area, risk of secondary surgery ranged from 4% (CI 1–9) in distal humerus fractures to 69% (CI 66–73) in hand fractures. For reoperations, the risk ranged from 4% (CI 1–9) in distal humerus fractures to 68% (CI 65–72) in hand fractures. For major reoperation the risk ranged from 2% (CI 0–6) in distal humerus fractures to 26% (CI 19–33) in fractures of the foot.

**Table:** Risk of secondary surgery, reoperation and major reoperation presented with 95% confidence intervals (CI) overall, by age, sex, and anatomical area overall using death as competing event estimated at 2 years following primary fracture-related surgery

Factor	n	Secondary surgery % (CI)	Reoperation % (CI)	Major reoperations % (CI)
All	9,719	20 (19–21)	19 (18–20)	8 (7–8)
Sex				
Male	3,597	24 (23–25)	23 (22–24)	8 (7–9)
Female	6,122	17 (17–18)	16 (15–17)	7 (7–8)
Age				
20–39	1,117	43 (40–46)	41 (38–44)	8 (7–10)
40–59	1,898	31 (29–33)	30 (28–32)	9 (7–10)
60–79	3,829	18 (16–19)	17 (16–18)	9 (8–10)
≥80	2,875	7 (6–8)	6 (5–7)	5 (4–6)
Anatomical area				
Shoulder	438	22 (18–26)	21 (17–25)	6 (4–9)
Humerus shaft	104	11 (6–18)	9 (4–15)	7 (3–13)
Distal humerus	102	4 (1–9)	4 (1–9)	2 (0–6)
Proximal antebrachium	313	37 (32–43)	35 (30–41)	10 (7–13)
Antebrachium shaft	101	15 (9–23)	14 (8–22)	7 (3–13)
Distal radius	1,724	8 (7–10)	8 (7–9)	3 (3–4)
Hand and fingers	657	69 (66–73)	68 (65–72)	5 (4–7)
Hip	3,931	11 (10–12)	10 (9–11)	8 (7–9)
Femur shaft	167	8 (5–13)	8 (4–13)	5 (2–9)
Distal femur	159	16 (11–22)	16 (11–22)	10 (6–15)
Patella	88	50 (39–60)	48 (37–58)	6 (2–12)
Proximal lower leg	293	32 (26–37)	27 (22–32)	6 (4–9)
Lower leg shaft	188	24 (18–31)	23 (18–30)	6 (3–10)
Ankle	1,290	29 (27–32)	28 (26–31)	11 (10–13)
Foot and toes	164	39 (32–46)	38 (31–46)	26 (19–33)

CI = 95% confidence interval.

### Sub-analysis

In order to estimate the issue of non-matching laterality, we performed a sub-analysis, in which we did not consider cases of secondary surgery with missing laterality for surgery in the DNPR. All occurrences with missing laterality of secondary, musculoskeletal surgery (n = 729), reoperations (n = 699), and major reoperations (n = 304) were not considered as secondary procedures. We then matched both personal identification numbers and laterality for primary and secondary surgeries. Risk of secondary surgery was 17% (CI 16–18), for reoperation it was 15% (CI 15–16), and for major reoperation it was 7% (CI (7–8).

## Discussion

This retrospective cohort study evaluated a large, nationwide cohort of 9,719 patients who had undergone primary, fracture-related surgery. We found an overall risk of experiencing any secondary musculoskeletal surgery 2 years after primary surgery of 20% (CI (19–21). For reoperation, the risk was 19% (CI 18–20) and for major reoperation, it was 8% (CI 7–8). The most important take-away message is that the risk of experiencing a major postoperative complication that needs surgical treatment is below 10%, after fracture-related surgery of the extremities.

### Risk of secondary procedures in fracture-related surgery

Overall, the risk of 20% for secondary surgery is comparable to other studies. Males had a slightly higher risk of undergoing secondary surgery and reoperations, however; when considering major reoperations, the risk was comparable to females. The reason for higher incidence of secondary surgeries in males is not evident from our study. An explanation could be a higher representation of males in the subgroups with more secondary surgeries, i.e., fractures of the hand, in which planned secondary procedures are common. When revisiting our data, men were indeed overrepresented in secondary surgeries for hands and fingers, constituting 71% of patients undergoing secondary surgeries. No other study considering reoperations for extremity fractures overall was identified, making direct comparison difficult. Only Pincus et al. have described a difference in risk of secondary surgery according to sex, in fractures of the ankle [4].

We noted that secondary surgeries and reoperations were more frequent among those aged 20 to 59, almost halving at ages 60 to 79, and diminishing considerably in patients aged above 80 years. However, for major reoperations, the distribution of risk is evenly distributed across ages. This could be explained by changes in fracture sites across different age groups or by surgeon unwillingness to perform secondary surgeries not prompted by a serious complication in elderly patients with higher surgical risks.

Findings from the existing literature for both upper and lower extremity fractures are comparable to ours. Bergdahl et al. [5] reported 17% risk of reoperation in fractures of the proximal humerus. Considering distal humeral fractures, a systematic review of the literature conducted in 2021 reported an incidence of 21% [19]. For distal radius fractures, a 2017 systematic review reported hardware removal in 9% [20]. A study considering fractures of the entire tibia on a large, non-select cohort reported a risk of reoperation of 30% [6]. For tibial plateau fractures the risk was 40% [7]. For fractures of the ankle, a Canadian study found that the risk of reoperation was 20% [4]. We report a considerably lower risk of reoperation of 4% for distal humerus fractures, which could be explained by the fact that only open reduction and internal fixation (ORIF) of complex intra-articular distal humerus fractures were included in the study.

We noted a high incidence of major reoperations was present in fractures of the foot and toes, reaching 26%. No apparent explanation can be found in our data. One may contemplate anatomical site in traumatological terms, containing several potential sites of fractures with risk of complicated healing. Furthermore, immobilization of the lower extremity can be problematic for patients to comply with.

### Secondary surgery vs reoperation and major reoperation

The methodological difference between secondary surgery, reoperations, and major reoperations is best exemplified in fractures of the hand and fingers and patella, in which K-wires are routinely used for fracture management. All these sites showed a high risk of secondary, musculoskeletal surgery but a notably lower risk of major reoperation. This is likely due to the use of K-wires, which are always removed in secondary procedures, naturally leading to an inflation of secondary, musculoskeletal surgery without it pertaining to a postoperative complication. Infections due to biofilm on implants can, however, occur several months and even longer after primary surgery and their treatment would not be classified as a major reoperation in our study unless the implant was removed. However, considering the size of our population and the relative rarity of these late complications, we believe our results to be representative of the true risk of complications warranting major reoperations.

### Bilateral observations

As addressed by Varnum et al. [21], the issue of bilateral observations can potentially violate the assumption of independence in using the cumulative incidence function. However, as also noted by Ranstam et al. [22], the issue seems to be minor when the outcome is secondary surgical procedures in large cohorts. We chose to present the risk of secondary procedures when not matching laterality of primary surgery with that of the secondary procedure. In order to assess the issue of missing laterality in the DNPR, we conducted a sub-analysis. We believed it more likely that a surgeon did not take the time to address laterality of the surgical procedure than that the patient had a contralateral fracture in the same area within 2 years. Although the true risk is somewhere in between the estimates presented in the primary and sub-analysis, we believe it closer to our primary results.

### Strengths and limitations

This study employed a large, nationwide cohort of patients. The homogeneity of the Danish population across regions and access to healthcare ensures high external validity [23]. Furthermore, we provide estimates of risk including both planned secondary surgeries and major reoperations using an outcome measure considered to be most appropriate in counselling patients [18].

Registration in the DFDB was not automated; rather it depended on the manual registration by surgeons, resulting in a rather low completeness of the DFDB in 2016 [13]. If fractures only managed by few and select hands were not registered due to individual choices by these surgeons, we would see an underrepresentation of these patients in the database. However, as fractures are usually treated by orthopedic surgeons of varying educational level and at many hospital sites, the external validity for only the most complex of fractures should potentially be at risk, as the most complex fractures are operated on exclusively at university hospital level. Likewise, if entire hospitals were to refuse registration, we could also see this selection bias. In the completeness study, however, we saw similar degrees of completeness across both small- and large-volume hospitals [13]. The true reason for the lack of completeness, however, is unknown.

In this study, we defined secondary surgery and reoperations by using specific codes and time limitations. This means that there is a risk of information bias in potential misclassifications when identifying relevant surgical procedures. For example, we did not include secondary surgeries coded with Q* surgical procedure codes (surgeries of the skin), which could be used for secondary surgery in patients with infection. However, reasons for secondary surgery, reoperations, and major reoperations were not elucidated. Furthermore, the distribution of fracture types according to classification systems such as AO were not available.

### Conclusion

In this nationwide register study on 9,719 adult patients surgically treated for fractures of the extremities, we provide accurate estimates of risk for any secondary, musculoskeletal surgery (20%, [CI 19–21]) as well as reoperations (19% [CI 18–20]) and major reoperations (8% [CI 7–8]) within 2 years of primary surgery.

## Notes

All authors contributed to conceiving this study. ABR wrote the manuscript. SJJ and ABR collected data and performed statistical analysis. All authors contributed to critical appraisal of the manuscript.

Handling co-editor: Bart A. Swierstra

*Acta* thanks Hans E Berg and Ville Mattila for help with peer review of this manuscript.
